# Primary Malignant Melanoma of The Endocervix Uteri and Outpatient Hysteroscopy as a Diagnostic Tool: Case Report and Literature Overview

**DOI:** 10.3390/diseases12060126

**Published:** 2024-06-09

**Authors:** Davide Dealberti, David Bosoni, Federica Spissu, Carla Pisani, Corinna Pizio, Luigi Nappi, Felice Sorrentino, Stefania Carlucci, Guglielmo Stabile

**Affiliations:** 1 Department of Obstetrics and Gynecology, Azienda Ospedaliera Universitaria SS. Antonio e Biagio e Cesare Arrigo, 15121 Alessandria, Italy; ddealberti@ospedale.al.it (D.D.); dd.bosoni@gmail.com (D.B.); cpisani@ospedale.al.it (C.P.); 2 Department of Gynecology and Obstetrics, University Hospital Maggiore Della Carità, School of Gynecology and Obstetrics, University of Eastern Piedmont, 28100 Novara, Italy; federica.spissu@outlook.it; 3 Department of Anatomic and Hystologic Pathology, Azienda Ospedaliera Universitaria SS. Antonio e Biagio e Cesare Arrigo, 15121 Alessandria, Italy; corinna.pizio@ospedale.al.it; 4 Department of Medical and Surgical Sciences, Institute of Obstetrics and Gynaecology, University of Foggia, 71122 Foggia, Italy; luigi.nappi@unifg.it (L.N.); felice.sorrentino.1983@gmail.com (F.S.); s.carlucci86@gmail.com (S.C.)

**Keywords:** melanoma of the cervix, endocervix, hysteroscopy, malignant neoplasia

## Abstract

Mucosal malignant melanoma has a low incidence and only 2% are diagnosed in the gynecological tract. Diagnosis of primary cervical malignant melanoma is often challenging. The clinical presentation mimics other malignant cervical tumors, usually with abnormal bleeding or discharge. Cervical screening tests, such as cytology, often fail to detect malignant melanomas because of the rarity of the disorder, and histological evaluation of lesions is of paramount importance. The treatment is often based on regimens used for cutaneous malignant melanoma. We present the first case in the literature of primary malignant melanoma of the endocervix diagnosed by outpatient hysteroscopy and we have performed a narrative review of the literature on PubMed, Scopus and Web of Science from 1980 to December 2023, identifying 137 cases. The most common initial symptom was vaginal bleeding in 82.8% of cases, and 84.8% of patients were menopausal at the time of diagnosis. The first diagnostic modality was biopsy in 67.7%; 90% of patients underwent surgery and 64.5% of the deaths occurred within the first 12 months after diagnosis. Primary malignant melanoma of the cervix is extremely rare and difficult to diagnose at an early stage which is due to the aggressiveness of the disease and the non-specificity of the symptoms. To improve survival, early diagnosis is essential and hysteroscopy could be a useful tool in achieving it. It is crucial to increase the attention of gynecologists on primary malignant melanoma of the cervix to also perform a diagnostic hysteroscopy in case of doubtful symptoms.

## 1. Introduction

Mucosal malignant melanoma (MM) has a low incidence and only 2% are diagnosed in the gynecological tract [1] Primary MM of the cervix are rare with around 137 cases described nowadays [2,3,4,5]. MM is a tumor derived from melanocytes [6]. Normal melanocytes have limited growth, derive from the neural crest and are largely distributed in all skin surfaces and most mucous membranes. Accordingly, MM appears mainly in the skin and mucous membranes, with a preference for areas exposed to ultraviolet radiation [4]. Even if MM is a rare skin cancer, it presents a high mortality rate, indicative of its aggressive nature [7]. More rarely, MM may arise from extra-cutaneous anatomical sites. One of these is represented by the female genital tract, with 5% of MM in female patients arising from the reproductive tract [8]. Diagnosis of primary cervical MM is often challenging. The clinical presentation mimics other malignant cervical tumors, usually with abnormal bleeding or discharge [9]. They may have an initial asymptomatic growth until the lesions eventually ulcerate. Cervical screening tests, such as cytology, often fail to detect malignant melanomas because of the rarity of the disorder and because they are very operator dependent [10]. Thus, histological evaluation of lesions is of paramount importance, and immunohistochemical staining proves very useful for the differential diagnosis of lesions. It is critical to exclude that the lesion is metastatic. Other primary sites, such as cutaneous, other mucosal, or ocular, should be excluded. Staging is also relevant to prognosis and treatment. Most authors use the Fédération Internationale de Gynécologie ed d’Obstétrique (FIGO) staging system for cervical cancer, which is also considered a prognostic factor for cervical MM [11]. Other prognostic factors described in the literature, such as lymph vascular space involvement, tumor thickness, lymph node status and neovascularization, are still debated. With regard to therapeutic strategy, there are no established guidelines and treatment is based on regimens used for cutaneous MM or the medical and surgical approaches similar to that used for squamous cell cervical carcinoma. The postoperative treatment is often based on the experience of the individual center or operator [12,13]. In previous years, radiotherapy and chemotherapy have been proposed as adjuvant therapies in association with various surgical treatments, such as radical hysterectomy and pelvic lymphadenectomy [11]. Alternative treatment options involve the use of monoclonal antibodies directed against cytotoxic T-lymphocyte-associated antigen 4, programmed cell death 1 and small-molecule inhibitors of the MAPK signaling pathway [14]. Several articles in the literature underline the efficacy of the anti-PD-1 antibody drug nivolumab in primary cervical and vaginal MM [15]. The aim of this study is to present the first case of diagnosis in the literature of primary malignant melanoma (MM) of the endocervix obtained by outpatient hysteroscopy and to perform a narrative review of the literature.

## 2. Materials and Methods

**Figure 1:**
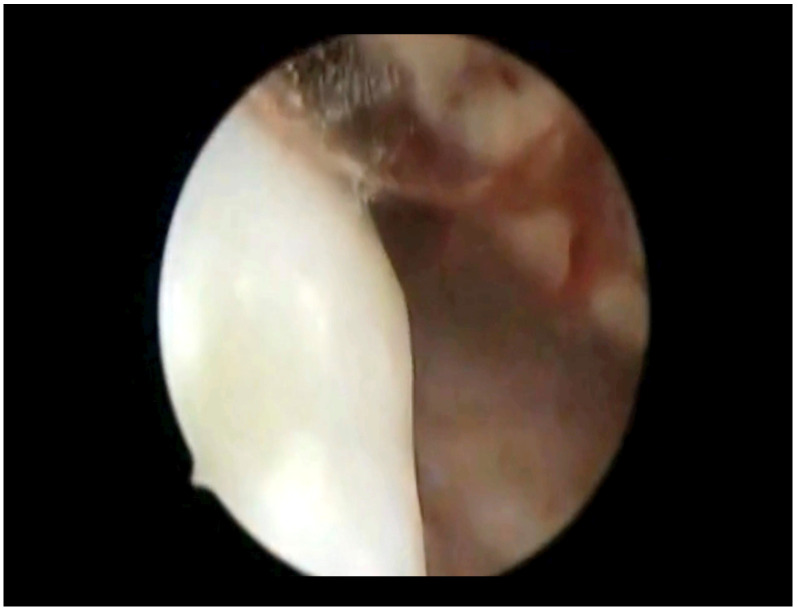
Hysteroscopic view: melanoma (whitish “snowflake-like” swelling).

**Figure 2:**
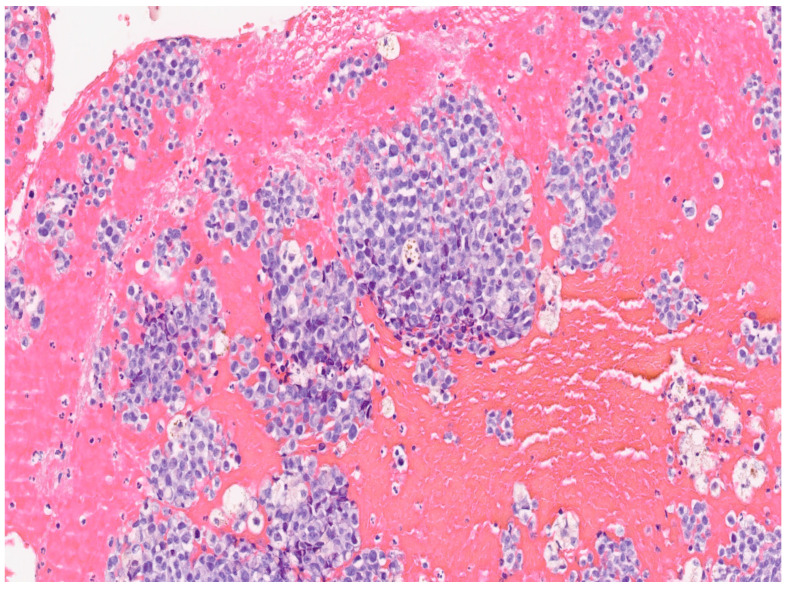
Histological examination of the endocervical biopsy. Microscopic magnification: 20×.

**Figure 3:**
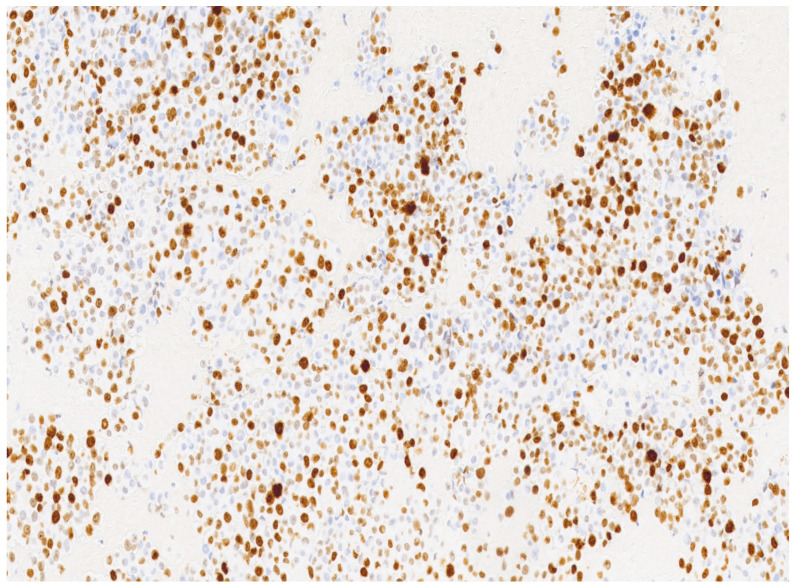
The proliferating fraction, assessed with monoclonal antibody Ki 6. Microscopic magnification: 20×.

**Figure 4:**
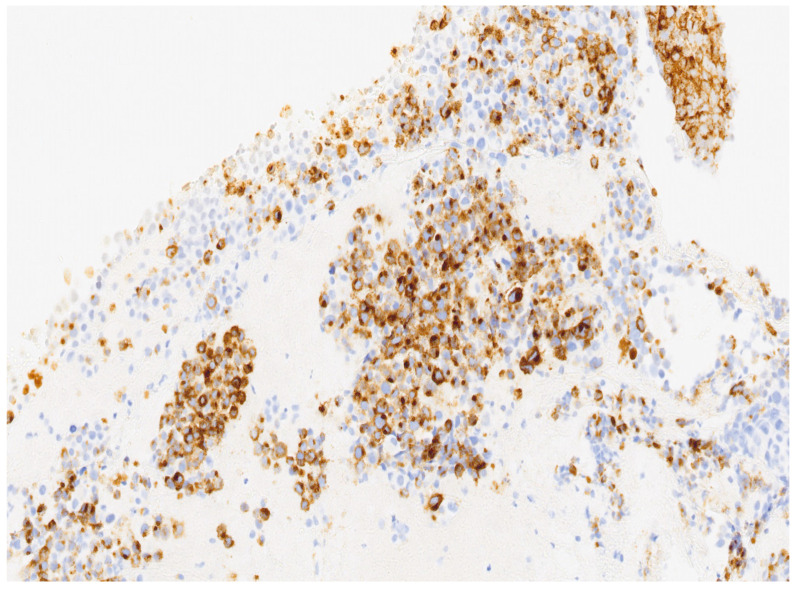
The cellular immunophenotypic profile resulted positive for antimelanoma HMB45. Microscopic magnification: 20×.

In our case, a 69-year-old nulliparous woman presented at the Department of Obstetrics and Gynecology, Azienda Ospedaliera Nazionale SS. Antonio e Biagio e Cesare Arrigo, Alessandria, Italy, complaining of postmenopausal abnormal uterine bleeding and pelvic pain. A few weeks before, she underwent a Pap test which showed Atypical Glandular Cells (AGC). Her medical history was consistent with arterial hypertension, well controlled by oral therapy. Her gynecological history included a revision of the uterine cavity 5 years earlier for an endometrial polyp. Gynecological examination showed normal vulva, vagina and ectocervix. The parameters were nodular, the cervix barrel-dilated, the uterus fixed. A transvaginal ultrasound revealed a vascularized endocervical lesion (color score 2) with a maximum diameter of 4 cm. Endometrial thickness was eight millimeters. Adnexa were regular according to the postmenopausal status. We performed hysteroscopy in an outpatient setting (without anesthesia or analgesia) with a 5.5 mm hysteroscope (Karl Storz endoscope). Vaginoscopic approach showed an atrophic ectocervix and a punctiform external uterine orifice with active bleeding at the entrance of the cervical canal. After synechiolysis and repeated washings, a whitish “snowflake-like” swelling occupying the right lateral wall of the caudal part of the endocervix was visualized (Figure 1). In the cranial part of the endocervix, there was a suspicious friable bleeding necrotic tissue, also suspicious in a heteroplasic sense. The isthmus appeared free from lesions. Inside the cavity, we were able to observe a cystic-gland endometrium, and a small endometrial polyp of about 1 cm. Multiple endocervical biopsies with micro scissors and forceps, and a complete excision of the endometrial polyp were carried out. The procedure lasted 12 min and there were no complications. Reported pain, measured with a Visual Analogue Scale (VAS) ranging from 0 to 10, was 2. The histological examination of the endometrial polyp was totally negative, while in the endocervical biopsies’ histological examination was reported the presence of multiple tissue fragments in which a neoplastic lesion with a solid growth pattern was present (Figure 2). Occasional focal accumulations of blackish material collected in coarse clumps referable to melanic pigment were observed. The proliferating fraction, assessed with monoclonal antibody Ki 67, was above 70% (Figure 3). The cellular immunophenotypic profile resulted positive for S100, antimelanoma HMB45 (Figure 4), tyrosinase and vimentin, suggesting a melanocytic cell origin of the lesion. Thus, the histomorphologic and immunohistochemical findings were considered consistent with a malignant melanoma. Determination of the mutational status of the BRAF gene and the N-RAS gene was negative. No mutations were detected in codon V600 of the BRAF gene and in codons 12, 13, 58, 59, 61, 117 and 146 of the N-RAS gene. The presence of primary melanoma in other more common sites at the time of diagnosis was ruled out. An extensive dermatological examination resulted completely negative. Within 15 days, the patient underwent a staging CT-PET scan total body and magnetic resonance image, which showed a voluminous expansive formation of the cervix of 75 × 76 × 66 mm, tightly adherent to the bladder without a secure plane of cleavage, initial infiltration of uterine vaginal septum and of lateral parameters, especially on the right, and intraperitoneal solid tissue tokens up to 15 mm in maximum size at the subhepatic site referable to peritoneal carcinosis. With these data, the patient was staged using the Fédération Internationale de Gynécologie et d’Obstétrique (FIGO) staging system for cervical cancer to FIGO stage IV B, therefore, after multidisciplinary discussion, was referred for immunotherapy with Pembrolizumab. Twelve days after the first administration of the monoclonal antibody, multiple subcutaneous nodulations appeared in the thoracic region. The right and left subclavicular lesions were biopsied (needle aspiration) with a cytologic result indicative for a melanocytic origin of the neoplasia. At the time of the second administration of Pembrolizumab, the patient reported radiating epigastric pain, and her blood analysis showed increased values of amylase and lipase. It was therefore decided to postpone immunotherapy and refer the patient to abdomen ultrasound and subsequent CT, which resulted positive for edematous pancreatitis. During hospitalization, the patient underwent therapeutic fasting, hydration and antibiotic therapy with Teicoplanin and Meropenem with gradual improvement of clinical and laboratory status. Metrorrhagia occurred during hospitalization, therefore, the patient was referred for palliative radiotherapy with hemostatic purposes. Two months after the start of palliative radiotherapy the patient deceased.

A search was carried out on PubMed, Scopus and Web of Science from 1980 to December 2023 to identify articles involving patients with cervical MM. The research was focused on original articles in English. A total of 137 manuscripts were detected through the research on databases and through references of the works. We have included in our review only articles in English. We have excluded articles and manuscripts not relevant for our review. We have used the following keywords: “Cervical Melanoma”, “Melanoma of the cervix”, “Diagnosis”, “Primary melanoma of the cervix”. Different combinations of the terms were used. Due to the rarity of this pathology, in the studies included there are many case reports. For this reason, we present the data in a descriptive manner.

## 3. Results

In our review, we identified a total of 137 cases of primary MM of the cervix uteri. The mean age of the patients was 56.5 years; 83 of these patients were in menopause. In 83% of the cases, the vaginal bleeding was the initial symptom and the second was vaginal discharge in 12% of the cases. The diagnosis was obtained by biopsy in 67% of the cases and with citology in 18% of the cases. Data on the presence of metastasis are present only for 98 patients and 82 of these did not have metastasis at the moment of diagnosis (84%); 90% of the patients were managed surgically with hysterectomy, salpingo-oophorectomy with or without lymphadenectomy. Only 8% of the patients received radiotherapy and 7% received chemotherapy. Data about outcomes are present in the literature for 105 patients. Of these, 58% died (61/105); 64% of deaths occurred in the first year from the diagnosis. Analyzing outcomes and prognosis, patients who underwent surgical management (of any type) had a significant improvement in prognosis. The overall survival was similar in patients who received adjuvant chemotherapy or radiotherapy.

## 4. Discussion

A recent systematic literature review, analyzing three electronic databases from 1980 to June 2022, identified 96 reports, comprising 137 patients [5]. By December 2023, no further case reports have been published, so our patient would be the one hundred thirty-eighth case reported. In the manuscript of Kechagias et al. [5], the most common initial symptom was vaginal bleeding in 82.8% of cases (101/122), and 84.8% of patients were menopausal at the time of diagnosis (84/99). These data are congruent with our case. Given that 50% of cervical MM and 35% of vaginal MM lack melanin pigmentation, MM should be differentiated from other pigmented or amelanotic masses [16]. In these cases, MM can be confused with poorly differentiated urothelial or squamous cell carcinomas, sarcomatoid carcinoma or pleomorphic sarcoma [17]. The first diagnostic modality was biopsy (excisional or punch biopsy) in 67.7% (65/96) of patients, proving superior to cytology, which was often negative or non-specific (17.7%, 17/96). Biopsies, where reported, were performed during gynecological examination, colposcopic examination or following surgery. Of the 96 diagnostic biopsies examined, only the one in our report was obtained during outpatient hysteroscopy. This is probably due to the presence of an endocervical lesion, without the involvement of the external part of the cervix. However, this raises the question of whether hysteroscopy, now the gold standard for the diagnosis of endometrial pathologies, could be proven to be a useful and innovative tool in this field as already demonstrated for other types of cancer affecting the cervix [18,19]. In 75 of the 138 cases under review, the FIGO system was used for staging with the following result: 37.3% stage I, 36% stage II, 18.7% stage III and 8% stage IV. Ninety per cent of patients underwent surgery: hysterectomy (radical or total) with bilateral adnexectomy with or without lymphadenectomy and partial vaginectomy with at least 2 cm free surgical margins which was the most commonly considered approach (40%). Chemotherapy combined or not with radiotherapy has been used as the main treatment strategy in a limited number of patients. Radiotherapy can be used for patients with tumor residue, parametrial involvement or positive lymph nodes. Furthermore, external pelvic radiotherapy and brachytherapy could be a useful therapeutic tool for patients who are inoperable, especially for palliative therapy [20]. Traditional cytotoxic agents have shown limited benefit in the treatment of metastatic disease. In the last years, the outcomes of patients with cutaneous melanoma are improved thanks to the introduction of two distinct classes of drugs that are the monoclonal antibodies targeting the cytotoxic T lymphocyte-associated antigen 4 and the programmed cell death 1, and the small molecule inhibitors of MAPK signaling pathway (which act on patients with melanoma who have BRAF mutations). Unfortunately, mucosal melanoma presents activating BRAF mutations in 10% of the cases making these drugs useful in a low percentage of cases [14]. Also in our case, the BRAF mutation was absent. However, immunotherapy and biological therapy deserve more in-depth study, not only for MM of the cervix but also in cases of metastatic melanoma. According to the recent literature, immunotherapy appears to improve the survival of patients with metastatic melanoma. However, data on the efficacy of new treatments are based on small retrospective studies and single-center experiences [14]. A paper by D’angelo et al. [21] provides an analysis of six studies reporting a relevant improvement in progression-free survival and response-rate for nivolumab combined with ipilimumab compared with the two agents alone, with good tumor responses durable over time. However, there are no studies in the literature specifically evaluating the role of immunotherapy in metastatic melanoma of the female genital tract. In our case, taking into account the stage of the disease, the general clinical conditions of the patient and after multidisciplinary counseling with the patient, it was decided to carry out this type of therapy. Clinical outcome data were reported for 106 patients, of whom 62 died (58.5%, 62/106) and 44 survived (41.5%, 44/106); 64.5% of the deaths occurred within the first 12 months after diagnosis. These data underline the poor prognosis of this disease. The follow-up period for surviving patients was 4 to 193 months. Primary malignant melanoma of the cervix is very rare, so uncommon that it is considered the least prevalent malignancy that can affect the cervix. It is considered to originate from melanocytic cells present in the cervix [22]. The presence of these cells was previously confirmed by Osamura et al. [23]. Since primary malignant melanoma of the cervix is very rare, cervical metastases from other more common sites such as the skin, uveal tract, esophagus and anorectal region should be excluded before making the diagnosis [19]. Morris and Taylor established four criteria for the diagnosis of primary cervical melanoma: (1) absence of melanoma elsewhere in the body; (2) presence of junctional change in the cervix; (3) presence of melanin pigment in the normal cervical epithelium and (4) metastasis according to the pattern of cervical carcinoma [24].

The disease most commonly manifests with non-specific symptoms such as bleeding or vaginal discharge, as cervical lesions can easily ulcerate and/or become infected, and most patients are postmenopausal [1]. However, rare cases of asymptomatic patients with diagnosis obtained through abnormal cytology have been reported [25]. As found in a recent meta-analysis, diagnosis is mainly obtained by biopsy (either excisional or punch biopsy), followed by cytology, ultrasound, MRI and CT scan [5]. In the case hereby presented, cytology was non-specific (Pap test: AGC) and the ectocervix was apparently normal. Therefore, the diagnosis was obtained by biopsy with micro forceps and micro scissors during office hysteroscopy with a 5.5 mm mini-operative hysteroscope, which allowed simultaneous visualization of a suspicious lesion and multiple biopsies in a safe, quick and well-tolerated way. Hysteroscopy, used for the first time for the diagnosis of a cervical melanoma, is an endoscopic surgical technique that has evolved into an extremely relevant tool for evaluating diagnosis of uterine pathology. Many hysteroscopic procedures can be performed in an outpatient setting, thanks to the vaginoscopic approach and the availability of high-definition mini-hysteroscopes that, without compromising optical performance, make it a simple, safe and well-tolerated office procedure with reduced costs [26,27]. This procedure thus allows direct visualization of the entire uterine cavity and offers the possibility of biopsy of suspicious lesions that may elude dilation and curettage. Given the fact that nowadays hysteroscopy with biopsies is currently considered the gold standard for the diagnosis of endometrial pathologies, superior to dilatation and curettage [28], it may also prove to be critical in the future for the diagnosis of endocervical pathologies. However, further studies are needed in this area, given the small number of recorded cases. Cervical MM is considered to be independent of human papillomavirus [29]. In the early stages, the disease is limited to the cervical mucosa and then invades adjacent organs, such as the uterosacral ligaments, the vaginal fornix, vulva and the pelvic wall, following what happens in other malignant cervical cancers [30]. Lymphatic metastases also follow the typical drainage pattern of other cervical carcinomas [31]. Although initially the TNM system was considered for staging as relevant to MM of other sites [31], the Fédération Internationale de Gynécologie ed d’Obstétrique (FIGO) staging system for cervical carcinoma has now been more widely applied to cervical MM, as it correlates better with the pattern of spread and prognosis [12,32]. Treatment of MM of the cervix is based primarily on the surgical approach. A recent integrated analysis confirmed the positive effect of hysterectomy on survival [33]. Radical hysterectomy with bilateral salpingoovariectomy and pelvic lymphadenectomy constitute the main therapeutic management for early-stage tumors with para-aortic lymphadenectomy being optional. Primary surgery with a free surgical margin of at least 2 cm is recommended for early-stage tumors [12,34]. Kechagias et al. believe that radical hysterectomy is not superior to simple hysterectomy, but the finding is controversial because of the small number of patients included in the sample [5]. The role of pelvic lymphadenectomy on prolonging disease-free survival of patients is still a matter of debate; however, it is useful for more accurate staging and to place indication for adjuvant therapy [34]. Chemotherapy in combination or not with radiotherapy has been chosen as first-line treatment only in cases of advanced or unresectable disease [34]. Their role as adjuvant therapies remains controversial, although many authors have used them after surgery in cases of positive margins or parametrial infiltration [35]. Recently, immunotherapy and biologic drugs have been considered for the treatment of primary MM of the cervix. Monoclonal antibodies directed against cytotoxic T lymphocyte antigen 4 (CTLA-4), often used in combination with antibodies targeting programmed death ligand 1 (PD-1), BRAF, KIT, VEGF and MEK1/MEK2 mutations, would appear to improve survival [36]. Other studies recommend that anti-PD-1 is associated with better overall survival than anti-CTLA4 in both advanced or recurrent melanoma of the cervix [13]. Furthermore, this treatment is well tolerated with an overall manageable toxicity profile. Some studies have analyzed data of patients receiving combined radiation therapy and immunotherapy in the adjuvant setting of melanoma of the lower genital tract. The data about overall survival were good [37]. Chanal et al. [38] reported a case of a locally advanced melanoma of the vagina. The patient was initially treated with TKI imatinib and ipilimumab, without clinical response. After tumor progression, a second line of treatment with pembrolizumab was established, with partial response and an important reduction of the disease. In the literature, other manuscripts support the importance of immunotherapy for the overall survival in cases of metastatic melanoma [39,40]. For this reason, immunotherapy deserves more in-depth studies not only for primary cervical MM but also in the metastatic forms [32]. Wang et al. have recently proposed a standardized treatment approach for patients with primary cervical MM, shown in Figure 1, although there are no officially established guidelines yet [41]. Primary MM of the cervix is associated with a poor prognosis, especially if not diagnosed at an early stage. Based on a 2012 study, the 5-year survival rates for stage I, II, III-IV are 18.8%, 11.1% and 0%, respectively [2,19]. According to a 2014 study, the 5-year overall survival is about 10%, and 87.5% of patients died within 3 years of diagnosis [11]. A recent analysis, conducted in 2022, revealed a higher 5-year overall survival of 30% and a mortality rate of 65% within the first 3 years after diagnosis, probably due to early diagnosis and surgical management [32]. An even more recent literature review found out that the majority of death (64%) occurred within 12 months of diagnosis. The prognosis was better for patients who underwent surgery. The survival rate was found to be similar in patients who did or did not receive adjuvant chemotherapy or radiotherapy [5]. According to a recent comprehensive analysis of case reports, the mean survival times from stage I to stage IV were 36.5, 20, 10 and 6 months, respectively [13,42].

## 5. Conclusions

Primary malignant melanoma of the cervix is extremely rare and difficult to diagnose at an early stage which is due to the aggressiveness of the disease and the non-specificity of the symptoms. Since, in our case, the ectocervix showed no macroscopically evident abnormality, while the lesion lay in the endocervix, no diagnosis probably could have been achieved without the aid of hysteroscopy. Therefore, hysteroscopy with biopsies, in the same way that it has been proven to be more accurate than dilatation and curettage for the diagnosis of endometrial pathologies, could be considered extremely useful for the early diagnosis of endocervical malignancies of unknown origin. Although the prognosis of primary malignant melanoma of the cervix is dismal, there has been an improvement in the survival of affected patients in recent years, which is probably due to early diagnosis and improved surgical strategies. Therefore, it is crucial to increase the attention of gynecologists on primary malignant melanoma of the cervix, also in reporting original cases, for better understanding its presentation, to avoid misdiagnoses and improve its treatments. Furthermore, it would be desirable in cases where there are doubtful symptoms without evidence of cervical lesions on examination (for example, genital bleeding post menopause) to perform a diagnostic hysteroscopy in a short time. In conclusion, due to the rarity of this pathology, an international register should be created to evaluate the efficacy of different types of treatment. Furthermore, other studies are required to test safety and long-term outcomes of immunotherapy in metastatic patients, and a better knowledge of molecular, genetic and epigenetic factors involved in disease progression is advisable to better understand factors that can improve outcomes of these patients.

## Data Availability

The authors confirm that the data supporting the findings of this study are available within the article.
